# Phosphorylation of PD-1-Y248 is a marker of PD-1-mediated inhibitory function in human T cells

**DOI:** 10.1038/s41598-019-53463-0

**Published:** 2019-11-21

**Authors:** Kankana Bardhan, Halil-Ibrahim Aksoylar, Thibault Le Bourgeois, Laura Strauss, Jessica D. Weaver, Bethany Delcuze, Alain Charest, Nikolaos Patsoukis, Vassiliki A. Boussiotis

**Affiliations:** 1 Division of Hematology-Oncology, Beth Israel Deaconess Medical Center, Harvard Medical School, Boston, MA 02215 United States; 2 Department of Medicine, Beth Israel Deaconess Medical Center, Harvard Medical School, Boston, MA 02215 United States; 3 Cancer Center, Beth Israel Deaconess Medical Center, Harvard Medical School, Boston, MA 02215 United States; 4 Present Address: Antoine Lacassagne Cancer Institute of Nice, Medical University of Nice Sophia Antipolis, Sophia Antipolis, France; 5 Jounce Therapeutics, 780 Memorial Drive, Cambridge, Massachusetts United States

**Keywords:** T cells, Predictive markers

## Abstract

PD-1 is a target of cancer immunotherapy but responses are limited to a fraction of patients. Identifying patients with T cells subjected to PD-1-mediated inhibition will allow selection of suitable candidates for PD-1-blocking therapy and will improve the therapeutic success. We sought to develop an approach to detect PD-1-mediated inhibitory signaling. The cytoplasmic tail of PD-1 contains an immunoreceptor tyrosine-based inhibitory motif (ITIM) encompassing Y223 and an immunoreceptor tyrosine-based switch motif (ITSM) encompassing Y248, which is indispensable for interaction of SHP-2 and delivery of PD-1 inhibitory function. We generated an antibody specific for phosphorylated PD-1-Y248 and examined PD-1pY248^+^ (pPD-1) expression in human T cells. pPD-1 was upregulated by TCR/CD3 + CD28 stimulation and simultaneous PD-1 ligation. pPD-1^+^CD8^+^ T cells were identified in human peripheral blood and had impaired effector function. pPD-1^+^ T cells were also detected in tumor-draining lymph nodes of tumor bearing mice and in biopsies of patients with glioblastoma multiform. Detection of pPD-1^+^ T cells might serve as a biomarker for identification of T cells subjected to PD-1-mediated immunosuppression.

## Introduction

The PD-1: PD-L1/2 pathway serves as a checkpoint to limit T-cell-mediated immune responses and prevent autoimmunity. Both PD-1 ligands, PD-L1 and PD-L2, engage the PD-1 receptor and induce PD-1 signaling and associated T-cell “exhaustion” (T_EX_)^1–5^. By expressing PD-1 ligands on the cell surface and engaging PD-1-positive effector T cells, tumors can co-opt the PD-1 pathway to evade an immune response^6–8^. PD-1-blocking antibodies, either as monotherapy or in combination with other compounds, have been used to enhance anti-tumor immunity in several cancer types with an acceptable safety profile^9–12^ and together with antibodies targeting CTLA-4 have revolutionized cancer treatment^13^.

A key mechanism by which cancer limits the host immune response is by upregulating PD-1 ligands in the tumor microenvironment (TME) which mediate ligation of PD-1 on tumor-specific CD8^+^ T-cells, leading to the development of T_EX_ cells that are incapable of generating anti-tumor responses^14^. Under these conditions, therapeutic targeting of PD-1 pathway induces expansion of oligoclonal CD8^+^ TILs that recognize tumor neoantigens^15^, and selectively expands CD8^+^ memory T cells^16^. Despite the significant progress with PD-1-based immunotherapy, durable clinical responses are only observed in a fraction of patients. Thus, a central question in the field of immunotherapy is how to predict the clinical response to PD-1 blockade in order to identify suitable candidates for PD-1 immunotherapy. Studies have evaluated several markers for potential roles in shaping, or predicting therapeutic responses including PD-L1 and PD-1 expression pattern, genetic mutations and neoantigens in cancer cells, cancer epigenetics, effector T cell landscape, and microbiota but results remain inconclusive^14,17–19^. Although most studies have paid attention to the inhibition of CD8^+^ T cells by PD-1, recently it was reported that functional systemic immunity of CD4^+^ T cells is required for recovery of CD8^+^ T cell responses after blockade of the PD-1 pathway indicating that CD4^+^ T cells are key targets of PD-1-mediated inhibition^20^. Together these extensive studies indicate that identifying which patients will respond to anti-PD-1 therapy and developing criteria for selection of suitable candidates will significantly improve the therapeutic success.

We sought to develop an approach that allows the detection of PD-1-mediated inhibitory signaling. To this end, we generated an antibody specific for phosphorylated Y248 of PD-1, which is required for interaction with SHP-2 and delivery of PD-1-mediated inhibition^21–23^, and examined expression and function of PD-1pY248^+^ T cells. We found that pPD-1 is upregulated in primary human CD4^+^ and CD8^+^ T cells during stimulation via CD3 and CD28 and PD-1 co-ligation with PD-L1. A fraction of pPD-1^+^CD8^+^ T cells is identified in the peripheral blood of healthy individuals and displays impaired production of effector cytokines. pPD-1^+^CD8^+^ T cells were also detected in tumor-draining lymph nodes of tumor-bearing mice. Furthermore, pPD-1^+^ cells were identified in biopsies of patients with glioblastoma. Our results indicate that pPD-1 identifies T cells subjected to PD-1-mediated immunosuppression, and can be detected in the context of cancer. Detection of pPD-1^+^ T cells in biopsies of cancer patients might serve as a biomarker for identification of patients with T cells subjected to PD-1-mediated immunosuppression, who would be suitable candidates for PD-1-checkpoint immunotherapy.

## Materials and Methods

### Generation of PD-1pY248 antibody

The PD-1 peptide H2N-CVPEQTE[pY]ATIVF-Ahx-KKK conjugated to KLH was emulsified in complete Freud’s adjuvant was used as immunogen to raise the polyclonal rabbit antiserum 1.2 (Rockland). Rabbit sera was screened by ELISA and was selected for reactivity on PD-1 pY248 peptide and lack of reactivity to unphosphorylated peptide. Affinity purified antibody was used for western blot. For use in flow cytometry, the affinity purified antibody was conjugated using Antibody Conjugation kits for R-Phycoerythrin (R-PE) (Lightening-Link antibody labelling kit, Innova Biosciences 703-0000), PE-Cy7 (abcam ab102903) or Alexa Fluor 488 (Lightening-Link antibody labelling kit, Innova Biosciences 707-0010).

### Cells and antibodies

Peripheral blood mononuclear cells (PBMC) were prepared from leukopacks (platelet apheresis byproduct) obtained at Brigham and Women’s Hospital, Boston. Tissue biopsies were obtained from patients with glioblastoma multiform. All human participants, including donors of PBMC and donors of tissue samples, provided informed consent for study participation. A protocol has been approved at the respective Institutional Review Board. All methods were performed in accordance with the relevant guidelines and regulations. Mononuclear cells were isolated by Ficoll (Amersham-Pharmacia Biotech, Piscataway, NJ) gradient centrifugation. The following antibodies were used for staining of freshly isolated human T cells: CD4 (Biolegend, Clone: A161A1), CD8 (Invitrogen, Clone: RPA-T8; Biolegend, Clone: HIT8A), CD45RO (Biolegend, clone: UCHL1), CCR7 (Biolegend, clone: G043H7), PD-1 (Biolegend, clone EH12.2H7), PD-L1 (Biolegend, clone 29E.2A3), CD80 (Biolegend, clone 2D10), CXCR5 (Biolegend, clone J252D4). Cells were subsequently fixed using formaldehyde (1.5%) for 10 min at RT. After fixation, cells were permeabilized using chilled BD Phosflow™ Perm Buffer III (BD Biosciences, cat# 558050) and stained with fluorescently-labelled pPD-1 antibody. For staining of mouse T cells, the following antibodies were used: CD4 (efluor 610, Thermo Fischer, cat# 61-0042-82), CD8 (Alexa Fluor 488, BD Biosciences, cat# 557668), CD44 (APC-Cy7, BioLegend cat# 103028), CD62L (BV605, BioLegend cat# 104438), CXCR3 (APC, BioLegend, cat# 126512) and CXCR5 (BV421, BioLegend, cat# 145512). Flow cytometry data were acquired by using BD LSR Fortessa (BD Biosciences) and Beckman Coulter Gallios Flow cytometers (Beckman Coulter Life Sciences) and were analyzed by FlowJo software. For immunofluorescence studies in FFPE biopsy sections from patients with glioblastoma multiform, tissue sections were stained with primary anti-Iba1 goat anti-human antibody (abcam cat# 5076) followed by secondary donkey anti-goat cy3.5 antibody (abcam cat# 6950) and pPD-1 antibody 1.2 (this study).

### Cell cultures

For *in vitro* culture, CD3^+^ primary human T cells were isolated by negative selection using a Pan T cell isolation kit (Miltenyi Biotec). Freshly isolated CD3^+^ human T cells were cultured with either media alone, PD-L1-Ig alone or with anti-CD3 (100 ng/ml) and anti-CD28 (300 ng/ml) mAbs (Fitzgerald International) for 24 hours followed by addition of IgG or PD-L1-Ig (10 ug/ml)) for an additional 24 hours. Cultures of primary human T cells were performed in 37 °C/5% CO_2_ incubator in RPMI 1640 supplemented with 2 mM L-glutamine (Cellgro/Mediatech, Manassas, VA), 10% heat-inactivated fetal bovine serum (FBS) (Atlanta Biologicals, Flowery Branch, GA), 10 mM HEPES, 1 mM sodium pyruvate, 50 U/ml Pen/Strep (from Cellgro/Mediatech, Manassas, VA), and 15 µg/ml gentamycin (from Gibco/Invitrogen, Grand Island, NY). For assessment of cytokine production, primary T cells were stimulated as indicated and intracellular expression of IFN-γ and TNF-α was analyzed with intracellular staining using antibodies to IFN-γ (Biolegend, B27) and TNF-α (Biolegend, Mab11) after gating on PD-1^+^ or PD-1pY248^+^ cells.

Jurkat T cells were stably transfected with PD-1, and stable lines were generated by culture with 5 µg/ml blasticidin. Before use in experiments, Jurkat T cells were rested overnight at 37 °C in RPMI-1640 containing 2% FBS and primary human or mouse T cells were rested under the same conditions for 1 hour. For pervanadate treatment, Jurkat-PD-1 T cells (5 × 10^6^ cells/sample) were washed twice with PBS and resuspended in 800 ul of per-warmed (37 °C) PBS. Pervanadate was prepared by mixing 5 ml 1 mM sodium orthovanadate (Na_3_VO_4_) with 5 ml 0.1% hydrogen peroxide (H_2_O_2_) (both made in PBS) and incubating 15 min at RT. A total of 200 ul of the H_2_O_2_/Na_3_VO_4_ mixture were added to the cells and incubated at 37 °C for the indicated time intervals. Reaction was stopped by adding 0.5 ml cold PBS and placing on ice. Cells were washed in cold PBS and lysed in lysis buffer containing 50 mM Tris-HCl, pH 7.4, 150 mM NaCl, 2 mM MgCl_2_, 10% glycerol and 1% NP-40 supplemented with 2 mM sodium orthovanadate, 1 mM sodium fluoride, 1 mM phenylmethylsulfonyl fluoride (PMSF), and protease Inhibitor Cocktail (Thermo Scientific). Cell lysates were resolved by SDS-PAGE and then analyzed by Western blotting. When pervanadate-treated cells were used for flow cytometry, after incubation with pervanadate for the indicated time intervals, cells were resuspended in FACS buffer (PBS 1x supplemented with 10% FBS) and washed twice. Subsequently 1 × 10^6^ cells per sample were fixed using formaldehyde (1.5%) for 10 min at RT. After fixation, cells were permeabilized using chilled BD Phosflow™ Perm Buffer III (BD Biosciences 558050) and stained with fluorescently-labelled pPD-1 antibody.

### Mouse tumor experiments

For tumor implantation, 8-10 weeks old female or male C57BL/6 mice were used and 0.5 × 10^5^ murine colon carcinoma (MC-38) cells were injected subcutaneously in the right flank. At day 15–16, mice were euthanized and tumor draining lymph nodes as well as distal, non tumor draining lymph nodes were collected and analyzed by flow cytometry. All procedures were performed in accordance with National Institutes of Health Guidelines for the Care and Use of Animals and approved by the Institutional Animal Care and Use Committee (IACUC) at Beth Israel Deaconess Medical Center.

### Statistics

Statistical significance was determined by two-tailed Student’s t test. Statistical significance for comparison among three or more groups was determined by ANOVA (*p value < 0.05; **p value < 0.01; ***p value < 0.001).

## Results and Discussion

### Phospho-PD-1 1.2 antibody specifically recognizes phosphorylated Y248 in PD-1 cytoplasmic tail

**Figure 1:**
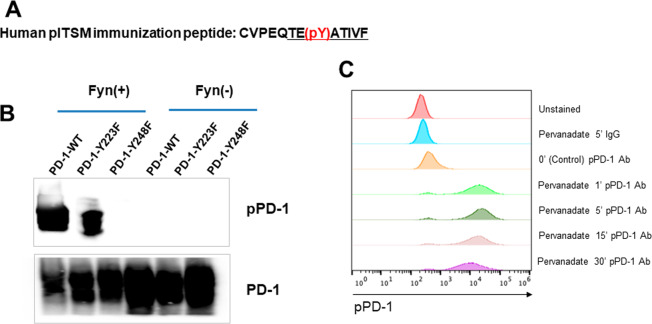
(**A**) Sequence of the conserved ITSM region in mouse and human PD-1 used in the immunogen peptide. The underlined region is conserved between mouse and human. (**B**) COS cells were transfected with cDNA for PD-1 and either kinase active or kinase inactive Fyn. Whole cell lysates were prepared 48 hours later and expression of the indicated proteins was assessed by immunoblot. (**C**) Jurkat T cells transfected with PD-1 were left untreated or incubated with pervanadate for various time intervals followed by incubation with PE-conjugated PD-1pY248 antibody or isotype control antibody.

It has been reported that SHP-2 may interact with both ITIM and ITSM of PD-1^24^ but association of SHP-2 with ITSM is required for PD-1 inhibitory function^21,22^. We generated an antibody (pPD-1 1.2) specific for phosphorylated ITIM Y248 in PD-1 cytoplasmic tail by using as immunogen a phosphotyrosine peptide of PD-1 ITSM, which is conserved between mouse and human (Fig. 1A). We have previously determined that TCR proximal Src family kinases can mediate PD-1 phosphorylation required for interaction with SHP-2^25^. To confirm specificity for phosphorylated PD-1, we co-transfected COS cells with human PD-1 cDNA together with kinase active or kinase inactive Fyn. PD-1 phosphorylation was detected in the presence of kinase active but not kinase inactive Fyn (Fig. 1B). To confirm specificity of pPD-1 1.2 antibody for PD-1pY248, we used cDNA of human PD-1 in which Y223 or Y248 was mutagenized to phenylalanine. Co-expression of kinase active Fyn together with human PD-1 WT or PD-1Y223F but not PD-1Y248F resulted in detection of PD-1 phosphorylation by pPD-1 1.2 Ab (Fig. 1C) confirming specificity for PD-1 Y248.

To examine whether pPD-1 Ab 1.2 would detect pPD-1 by flow cytometry, we used Jurkat T cells stably transfected with human PD-1 cDNA. To induce PD-1 phosphorylation we treated Jurkat-PD-1 cells with pervanadate, which was previously established to induce PD-1 phosphorylation required for interaction with SHP-2^22^. Pervanadate treatment induced PD-1 phosphorylation that was detected by flow cytometry using pPD-1 1.2 Ab (Fig. 1C).

### PD-1 phosphorylation is induced in primary human T cells during TCR/CD3 and CD28 stimulation and PD-1 ligation by PD-L1

**Figure 2:**
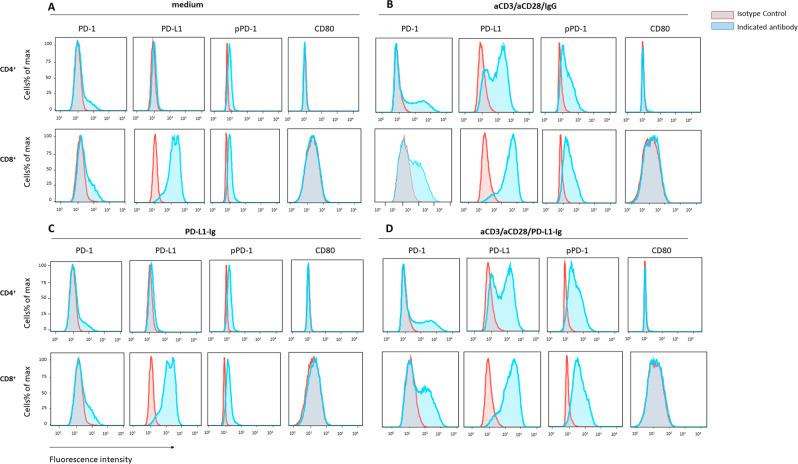
PD-1-Y248 phosphorylation is induced after stimulation of primary human CD4^+^ and CD8^+^ T cells. Purified T cells were left unstimulated (**A**) or cultured with aCD3/aCD28/IgG (**B**), PD-L1-Ig (**C**) or aCD3/aCD28/PD-L1-Ig (**D**) as indicated in Methods and after gating on CD4^+^ or CD8^+^ T cells expression of PD-1, pPD-1, PD-L1 and CD80 was examined. Results are representative of six independent experiments.

To examine regulation of PD-1 phosphorylation in primary human T cells, we used CD3^+^ T cells isolated from PBMC of healthy volunteer donors. Expression of PD-1, pPD-1, PD-L1 and CD80 was examined on CD4^+^ and CD8^+^ T cells during stimulation in the presence or in the absence of PD-1 ligation. Low level PD-1 expression and PD-1 phosphorylation was detected prior to stimulation *in vitro* (Fig. 2A). Stimulation of CD4^+^ T cells via TCR/CD3 and CD28 by using aCD3/CD28/IgG-coated beads upregulated expression of PD-1 and induced expression of PD-L1 and phosphorylation of PD-1Y248 whereas expression of CD80 was not detected (Fig. 2B). Co-ligation of PD-1 during stimulation of CD4^+^ T cells via TCR/CD3 and CD28 increased the intensity of pPD-1-Y248 expression (Fig. 2D) compared to stimulation without PD-1 ligation induced by aCD3/CD28 stimulation (Fig. 2B; Supplementary Fig. S1). A similar pattern of PD-1 expression and phosphorylation was observed in CD8^+^ T cells (Fig. 2A,B,D; Supplementary Fig. S1). Gating on cells that obtained PD-1 expression after stimulation with or without PD-1 ligation, showed that pPD-1 was expressed in the PD-1^+^ population of CD4^+^ or CD8^+^ subsets (Supplementary Fig. S2). Upregulation of PD-1 and PD-L1 and phosphorylation of PD-1 required signaling via TCR/CD3 and CD28 and were not induced by culture with PD-L1-Ig alone (Fig. 2C). These results indicate that engagement of the PD-1 pathway can be detected in human T cells by phosphorylation of PD-1Y248 using a site-specific antibody. Notably, compared to CD4^+^ T cells, CD8^+^ T cells expressed constitutively higher levels of PD-L1 prior to *in vitro* stimulation and a higher degree of baseline PD-1 phosphorylation (Fig. 2A).

### T cells expressing PD-1Y248 phosphorylation are detected in the peripheral blood of healthy individuals

**Figure 3:**
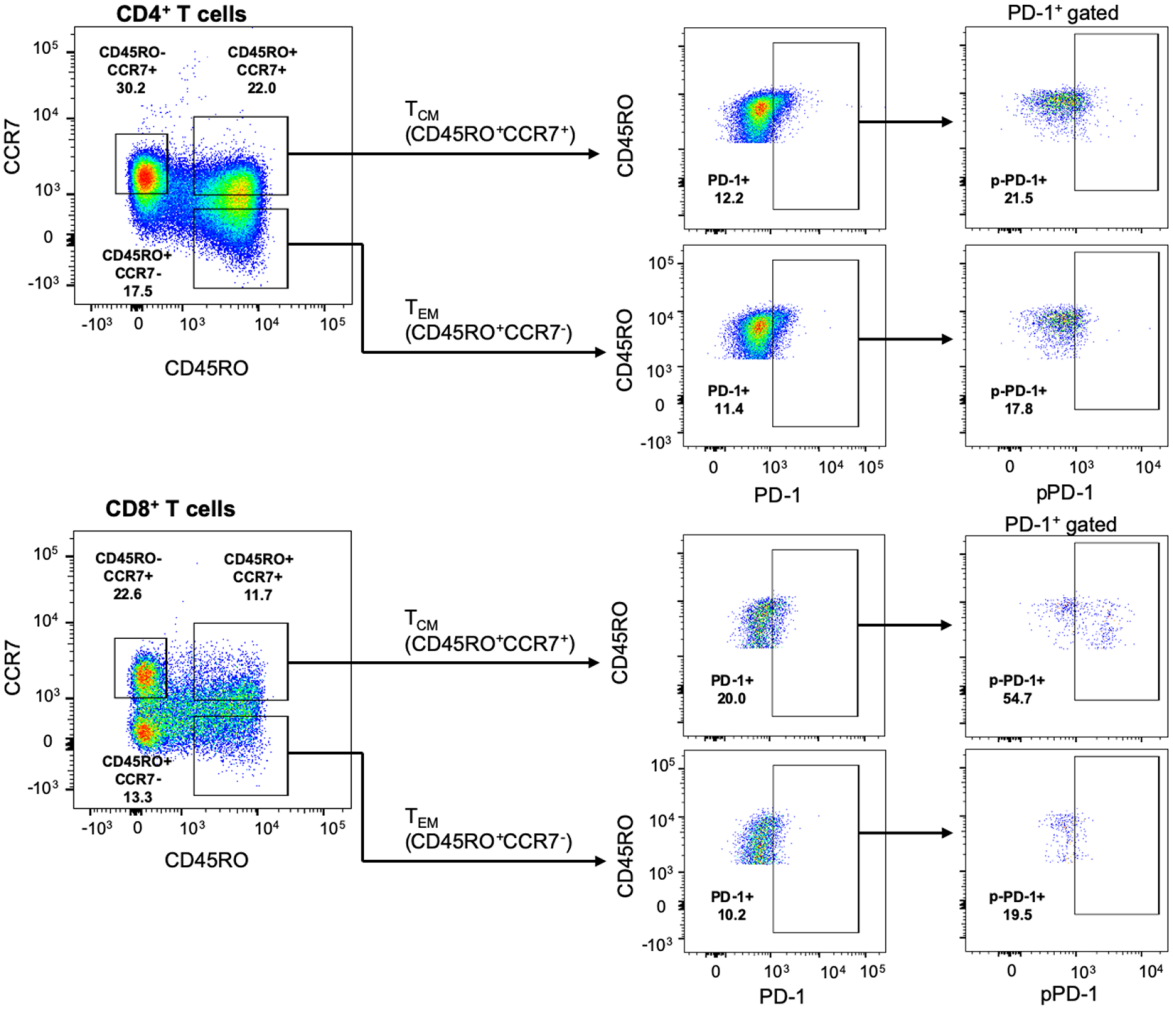
pPD-1 is predominantly expressed in CD8^+^ T_CM_ cells. CCR7 and CD45RO markers were used to identify central memory (T_CM_) and effector memory (T_EM_) T cells. After gating on CD45RO expression, T_CM_ (CD45RO^+^CCR7^+^) and T_EM_ (CD45RO^+^CCR7^−^) CD4^+^ and CD8^+^ T cells were identified by assessing expression of CCR7. In T_CM_ and T_EM_ populations, expression of PD-1 was determined and, subsequently, expression of pPD-1 (pPD-1-Y248) was assessed in the PD-1^+^ population within each subset. Results are representative of six separate experiments.

Because our studies showed that a small fraction of primary human T cells constitutively expressed PD-1pY248 (Fig. 2A), we sought to further characterize this population and its function. Using peripheral blood mononuclear cells from healthy donors we determined that primary T cells in the memory compartment expressed PD-1 (Fig. 3), consistent with previous observations^26^. Within this population, pPD-1^+^ cells could be detected within CD4^+^ but mostly within CD8^+^ T fraction, predominantly in the CD45RO^+^CCR7^+^ subset and to a significantly lower extent in the CD45RO^+^CCR7^−^ subset (Fig. 3; Supplementary Fig. S3), which represent central memory (T_CM_) and effector memory (T_EM_) subsets respectively^27^. Although the overall expression of pPD-1 in these non-stimulated peripheral blood T cells is low compared to *in vitro* stimulated T cells, it is noteworthy that higher pPD-1 expression level was detected in CD45RO^intermediate^ rather than CD45RO^hi^ cells, suggesting that pPD-1^+^ cells are possibly in the process of reducing CD45RO expression (Fig. 3).

**Figure 4:**
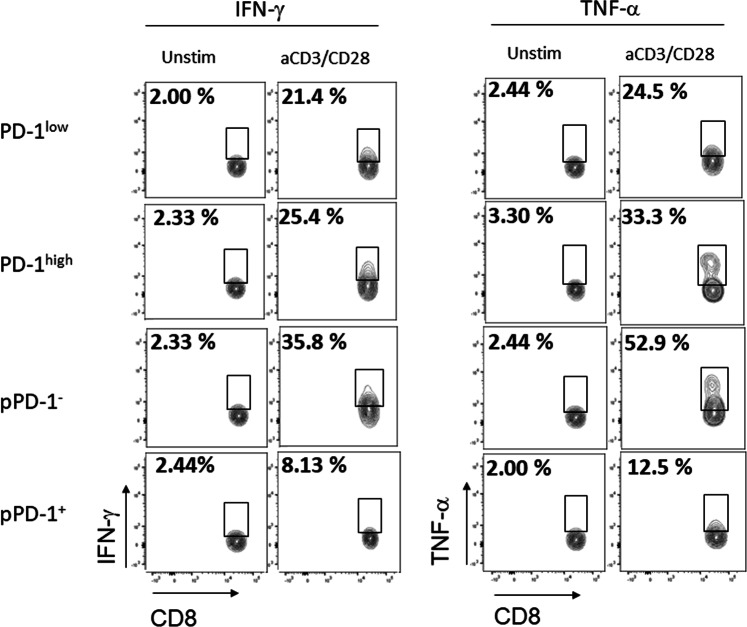
PD-1-Y248 phosphorylation but not PD-1 expression level inversely correlates with inhibition of CD8^+^ T cell effector function. Primary human CD8^+^ T cells were cultured with anti-CD3 and anti-CD28 for 6 hours and production of IFN-γ and TNF-α was examined by flow cytometry after gating on PD-1^hi^, PD-1^lo^ cells or after gating on pPD-1^+^ and pPD-1^−^ cells. Results are representative of three experiments.

Previously, it was determined that the expression level of PD-1 in peripheral blood of healthy individuals did not correlate with properties of T_EX_ cells or impaired cytokine production during stimulation *in vitro*^26^. Because phosphorylation of PD-1-Y248 is mandatory for PD-1-mediated inhibition^21–23^, we examined whether expression of pPD-1 in peripheral blood CD8^+^ T cells might correlate with impaired effector function. For this purpose, we assessed intracellular expression of IFN-γ and TNF-α in PD-1^+^ and pPD-1^+^ CD8^+^ T cells after stimulation *in vitro*. Although PD-1^high^ cells did not display diminished production of these effector cytokines, pPD-1^+^ CD8^+^ T cells had impaired expression of IFN-γ and TNF-α in response to stimulation *in vitro* (Fig. 4). Thus, PD-1pY248 is a marker of CD8^+^ T cells with impaired capacity to mount immune responses.

### CD8^+^CXCR5^+^ central memory human T cells express pPD-1

**Figure 5:**
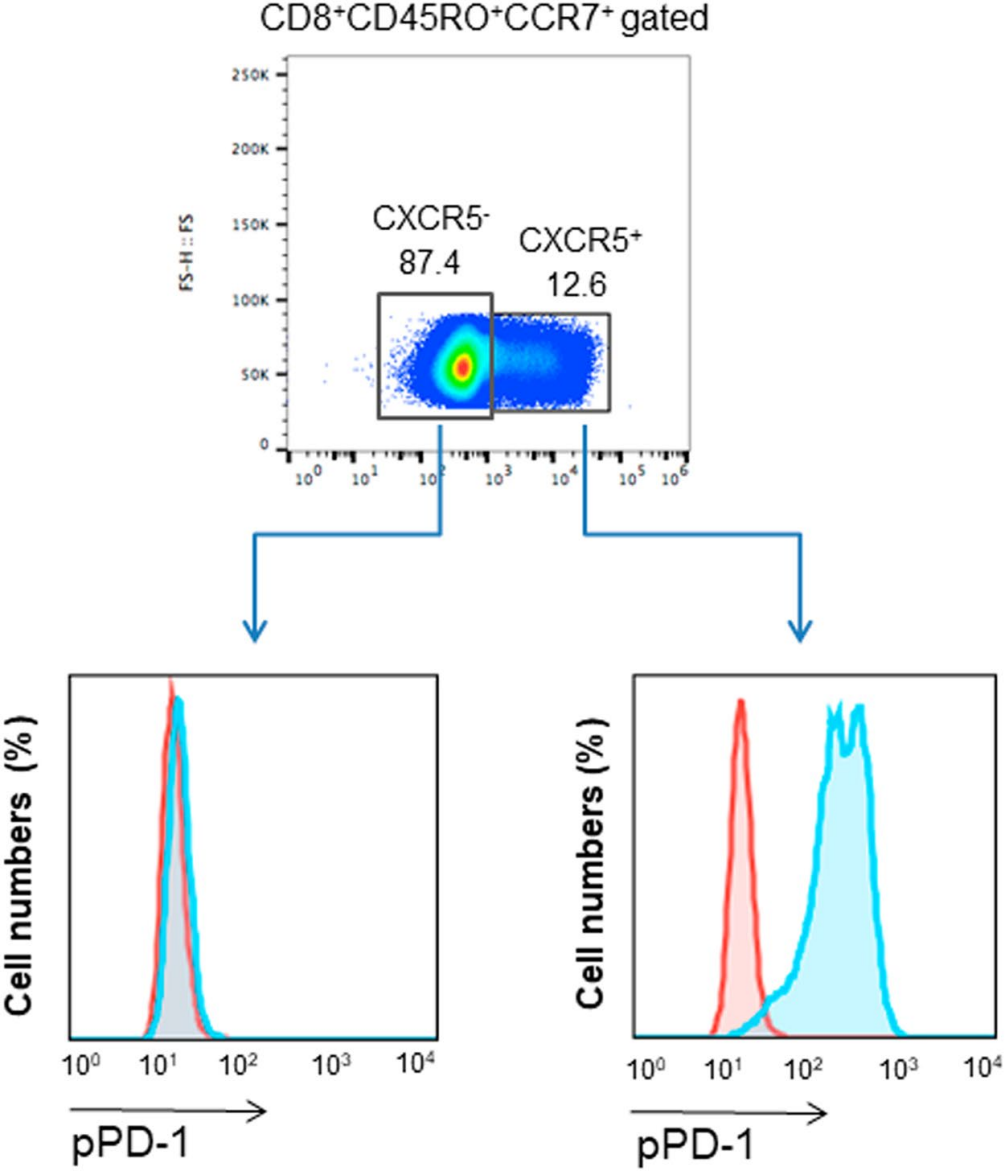
CD8^+^CXCR5^+^ central memory human T cells express pPD-1. After gating on CD8^+^CD45RO^+^CCR7^+^ T_CM_ cells as in Fig. 3, expression of CXCR5 was determined and, subsequently, expression of pPD-1 (PD-1-pY248) was assessed on CXCR5^+^ and CXCR5^−^ cells. Results are representative of six independent experiments.

We are intrigued by the presence of pPD-1^+^ cells, which displayed impaired effector responses, in the peripheral blood of healthy donors and we sought to examine their properties further. Recent studies determined that a subset of PD-1^+^CD8^+^ T_EX_ cells that are subjected to PD-1-mediated inhibition during chronic viral infection, expresses the chemokine receptor CXCR5^28,29^. Importantly, the proliferative burst induced after PD-1 blockade in such chronically infected mice comes exclusively from this CD8^+^ T cell subset^29^. In humans, such cell population is detectable during chronic infections (e.g. HIV) but also during acute infection and after immunization, and resides within the T_CM_ CD8^+^ subset^30^. For these reasons, we examined whether human CXCR5^+^ in the T_CM_ subset might express pPD-1. Assessment of pPD-1 showed that the vast majority of peripheral blood CXCR5^+^CD8^+^ T cells in the CD45RO^+^CCR7^+^ compartment expressed pPD-1 (Fig. 5). Together with our findings that pPD-1^+^ cells have impaired production of effector cytokines (Fig. 4), these results indicate that pPD-1 might identify a population of CD8^+^ T cells resembling the quiescent CD8^+^CXCR5^+^ T_EX_ cells that are subjected to PD-1-mediated inhibitory function. Furthermore, these data suggest that pPD-1 might serve as a marker of CD8^+^ T_EX_ cells.

### pPD-1 is expressed in the context of cancer in a mouse tumor model and patients’ biopsies

**Figure 6:**
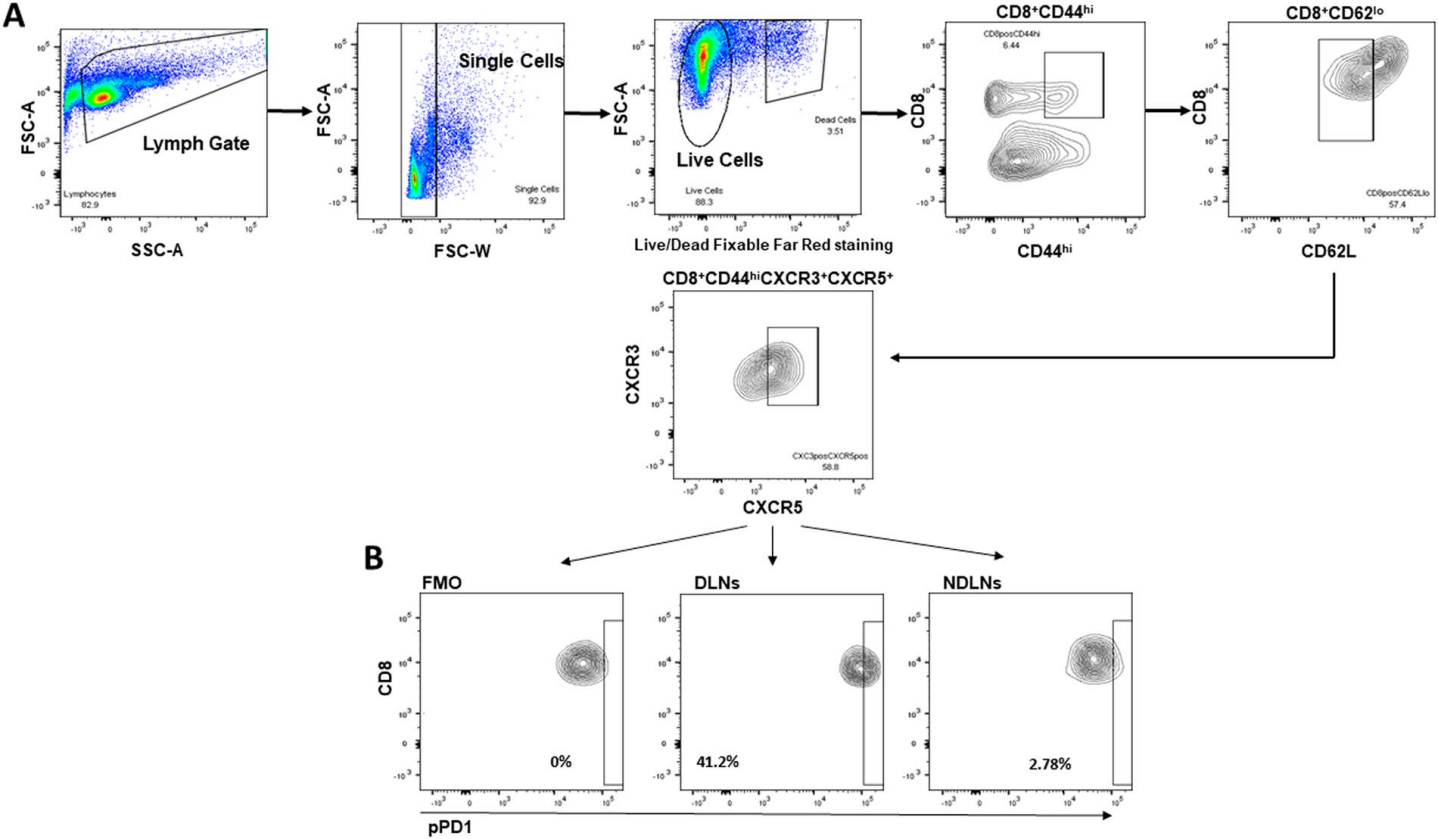
pPD-1 is expressed in T_FC_-like cells in tumor draining lymph nodes. C57BL/6 mice were inoculated with MC-38 colon adenocarcinoma tumor cells (5 × 10^5^/mouse) and 15 days later, after gating on CD8^+^CD44^hi^CXCR3^+^CXCR5^+^ T cells using the gating strategy shown in (**A**), expression of pPD-1 was assessed in the tumor draining lymph nodes (DLNs) and distant, non-tumor draining lymph nodes (NDLNs) (**B**).

To examine whether PD-1 phosphorylation is detected in the context of cancer, we used the MC-38 colon adenocarcinoma mouse tumor model. We examined whether pPD-1 was detected in T_FC_-like CD8^+^ cells, which are recruited to tumor draining lymph nodes (DLNs), express CD44 and CXCR3 and are subjected to PD-1-mediated inhibition^31^. We found that in tumor-bearing mice, CD44^high^CXCR3^+^CXCR5^+^CD8^+^ T cells expressing pPD-1 were detected in tumor DLNs but not in distal, non-tumor draining lymph nodes (NDLNs) (Fig. 6).

**Figure 7:**
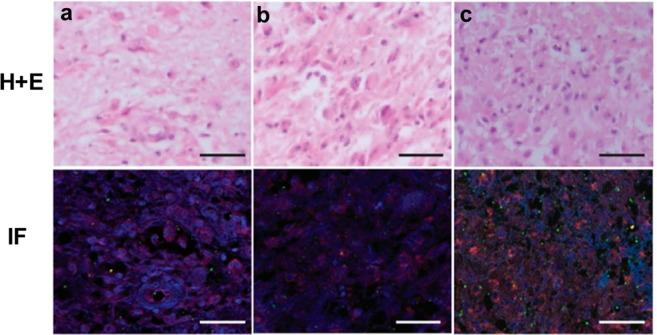
pPD-1 is detected in the microenvironment of human cancer. Representative H + E stain (**a**–**c**, top raw) and confocal photomicrographs of indirect immunofluorescence (bottom raw) of glioblastoma FFPE sections from three different patients co-stained for identification of the microglia/macrophage-specific ionizing calcium-binding adaptor molecule 1 (Iba1; red), pPD-1 (green), and DAPI (blue). Bar = 50 um.

To test whether pPD-1^+^ cells might be detected in the context of human cancer, we used biopsies from patients with glioblastoma. In three different patients, we detected expression of pPD-1^+^ cells in the tumor microenvironment (Fig. 7). Notably, different levels of pPD-1 expression are detected in these biopsies indicating that pPD-1 expression level might be an informative biomarker of PD-1-mediated inhibition in the context of cancer, which might vary among patients. Further studies will investigate the expression of pPD-1 and its significance in various cancers.

Our present studies showed that activation of the PD-1 pathway in human T cells can be identified by phosphorylation of PD-1-Y248, a site indispensable for induction of PD-1 inhibitory function^21–23^. Our findings point to the conclusion that assessing PD-1 phosphorylation in patients with chronic infections and cancer might serve as an indicator of PD-1 inhibitory function. For example, identification of pPD-1^+^ CD8^+^ T cells in tumor biopsies, might be informative for identifying patients who have evidence of PD-1-mediated inhibitory signaling and would be best candidates to be benefitted by immunotherapy with PD-1 checkpoint blockade. Because circulating CD8^+^ T cells contain high frequency of neoantigen-specific T cells within the PD-1^+^ fraction^32^, it will be important to determine whether such T cells reside preferentially within the pPD-1^+^ population in the peripheral blood of patients with cancer. Pathogen-specific CD8^+^ T cells subjected to PD-1 inhibitory signaling in chronic infections such as HIV, Hep C, CMV or EBV might also reside within the pPD-1^+^CD8^+^ population. Further studies will investigate the expression and function of pPD-1^+^ T cells in the context of cancer and chronic infections.

In our studies, we identified expression and function of pPD-1^+^CD8^+^ T cells in the peripheral blood of healthy individuals. PD-1 is a key mediator of peripheral tolerance^33^ and aberration in the function of this pathway leads to autoimmunity in several mouse models^34–38^. Similarly, a regulatory polymorphism in PD-1 gene in humans is associated with susceptibility to systemic lupus erythematosus^39^. Thus, pPD-1 detection in T cells of healthy individuals may serve as an indicator of self-tolerance, whereas lack or decrease of pPD-1 expression might serve as an indicator of autoimmunity.

In conclusion, we generated a new tool to assess PD-1-mediated inhibitory signaling in human T cells, which allows the identification of immune cells with impaired effector function *in vivo* and might serve as a biomarker to facilitate clinically relevant decisions for selection of suitable candidate patients for PD-1 checkpoint immunotherapy.

## Supplementary information

Supplementary Figures

## References

[1] Barber DL (2006). Restoring function in exhausted CD8 T cells during chronic viral infection. Nature.

[2] Day CL (2006). PD-1 expression on HIV-specific T cells is associated with T-cell exhaustion and disease progression. Nature.

[3] Petrovas C (2006). PD-1 is a regulator of virus-specific CD8+ T cell survival in HIV infection. The Journal of experimental medicine.

[4] Wherry EJ (2011). T cell exhaustion. Nature immunology.

[5] Boussiotis VA (2016). Molecular and Biochemical Aspects of the PD-1 Checkpoint Pathway. The New England journal of medicine.

[6] Latchman Y (2001). PD-L2 is a second ligand for PD-1 and inhibits T cell activation. Nature immunology.

[7] Dong H (2002). Tumor-associated B7-H1 promotes T-cell apoptosis: a potential mechanism of immune evasion. Nature medicine.

[8] Azuma T (2008). B7-H1 is a ubiquitous antiapoptotic receptor on cancer cells. Blood.

[9] Topalian SL (2012). Safety, activity, and immune correlates of anti-PD-1 antibody in cancer. The New England journal of medicine.

[10] Lipson EJ (2013). Durable cancer regression off-treatment and effective reinduction therapy with an anti-PD-1 antibody. Clin Cancer Res.

[11] Ansell SM (2015). PD-1 blockade with nivolumab in relapsed or refractory Hodgkin’s lymphoma. The New England journal of medicine.

[12] Garon EB (2015). Pembrolizumab for the treatment of non-small-cell lung cancer. The New England journal of medicine.

[13] Topalian SL (2015). Immunotherapy: The path to win the war on cancer?. Cell.

[14] Zou W, Wolchok JD, Chen L (2016). PD-L1 (B7-H1) and PD-1 pathway blockade for cancer therapy: Mechanisms, response biomarkers, and combinations. Science translational medicine.

[15] Rizvi NA (2015). Cancer immunology. Mutational landscape determines sensitivity to PD-1 blockade in non-small cell lung cancer. Science (New York, N.Y.

[16] Ribas A (2016). PD-1 Blockade Expands Intratumoral Memory T Cells. Cancer immunology research.

[17] Taube JM (2014). Association of PD-1, PD-1 ligands, and other features of the tumor immune microenvironment with response to anti-PD-1 therapy. Clin Cancer Res.

[18] Herbst RS (2014). Predictive correlates of response to the anti-PD-L1 antibody MPDL3280A in cancer patients. Nature.

[19] Tumeh PC (2014). PD-1 blockade induces responses by inhibiting adaptive immune resistance. Nature.

[20] Zuazo M (2019). Functional systemic CD4 immunity is required for clinical responses to PD-L1/PD-1 blockade therapy. EMBO molecular medicine.

[21] Okazaki T, Maeda A, Nishimura H, Kurosaki T, Honjo T (2001). PD-1 immunoreceptor inhibits B cell receptor-mediated signaling by recruiting src homology 2-domain-containing tyrosine phosphatase 2 to phosphotyrosine. Proceedings of the National Academy of Sciences of the United States of America.

[22] Chemnitz JM, Parry RV, Nichols KE, June CH, Riley JL (2004). SHP-1 and SHP-2 associate with immunoreceptor tyrosine-based switch motif of programmed death 1 upon primary human T cell stimulation, but only receptor ligation prevents T cell activation. J Immunol.

[23] Yokosuka T (2012). Programmed cell death 1 forms negative costimulatory microclusters that directly inhibit T cell receptor signaling by recruiting phosphatase SHP2. The Journal of experimental medicine.

[24] Sheppard KA (2004). PD-1 inhibits T-cell receptor induced phosphorylation of the ZAP70/CD3zeta signalosome and downstream signaling to PKCtheta. FEBS letters.

[25] Patsoukis, N. *et al*. Interaction of both SH2 domains of SHP-2 with a PD-1 homodimer is required for PD-1-mediated inhibition of T cell responses. *J Immunol May 2017, 198 (1 Supplement) 124.11* (2017).

[26] Duraiswamy J (2011). Phenotype, function, and gene expression profiles of programmed death-1(hi) CD8 T cells in healthy human adults. J Immunol.

[27] Sallusto F, Lenig D, Forster R, Lipp M, Lanzavecchia A (1999). Two subsets of memory T lymphocytes with distinct homing potentials and effector functions. Nature.

[28] He R (2016). Follicular CXCR5-expressing CD8+ T cells curtail chronic viral infection. Nature.

[29] Im SJ (2016). Defining CD8+ T cells that provide the proliferative burst after PD-1 therapy. Nature.

[30] Leong YA (2016). CXCR5(+) follicular cytotoxic T cells control viral infection in B cell follicles. Nature immunology.

[31] Chamoto K (2016). Mitochondrial activation chemicals synergize with surface receptor PD-1 blockade for T cell-dependent antitumor activity. Proceedings of the National Academy of Sciences of the United States of America.

[32] Schumacher TN, Scheper W, Kvistborg P (2019). Cancer Neoantigens. Annual review of immunology.

[33] Francisco LM, Sage PT, Sharpe AH (2010). The PD-1 pathway in tolerance and autoimmunity. Immunological reviews.

[34] Nishimura H, Nose M, Hiai H, Minato N, Honjo T (1999). Development of lupus-like autoimmune diseases by disruption of the PD-1 gene encoding an ITIM motif-carrying immunoreceptor. Immunity.

[35] Nishimura H (2001). Autoimmune dilated cardiomyopathy in PD-1 receptor-deficient mice. Science (New York, N.Y.

[36] Ansari MJ (2003). The programmed death-1 (PD-1) pathway regulates autoimmune diabetes in nonobese diabetic (NOD) mice. The Journal of experimental medicine.

[37] Salama AD (2003). Critical role of the programmed death-1 (PD-1) pathway in regulation of experimental autoimmune encephalomyelitis. The Journal of experimental medicine.

[38] Martin-Orozco N, Wang YH, Yagita H, Dong C (2006). Cutting Edge: Programmed death (PD) ligand-1/PD-1 interaction is required for CD8+ T cell tolerance to tissue antigens. J Immunol.

[39] Prokunina L (2002). A regulatory polymorphism in PDCD1 is associated with susceptibility to systemic lupus erythematosus in humans. Nature genetics.

