# Publisher Correction: Phosphorylation of PD-1-Y248 is a marker of PD-1-mediated inhibitory function in human T cells

**DOI:** 10.1038/s41598-020-71090-y

**Published:** 2020-09-23

**Authors:** Kankana Bardhan, Halil-Ibrahim Aksoylar, Thibault Le Bourgeois, Laura Strauss, Jessica D. Weaver, Bethany Delcuze, Alain Charest, Nikolaos Patsoukis, Vassiliki A. Boussiotis

**Affiliations:** 1 Division of Hematology-Oncology, Beth Israel Deaconess Medical Center, Harvard Medical School, Boston, MA 02215 USA; 2 Department of Medicine, Beth Israel Deaconess Medical Center, Harvard Medical School, Boston, MA 02215 USA; 3 Cancer Center, Beth Israel Deaconess Medical Center, Harvard Medical School, Boston, MA 02215 USA; 4 Present Address: Antoine Lacassagne Cancer Institute of Nice, Medical University of Nice Sophia Antipolis, Sophia Antipolis, France; 5 Present Address: Jounce Therapeutics, 780 Memorial Drive, Cambridge, MA USA

Correction to: *Scientific Reports* 10.1038/s41598-019-53463-0, published online 21 November 2019

In the original version of this Article, the author Thibault Le Bourgeois was incorrectly indexed. This error has now been corrected.

