# Supplementary material for: Phosphorylation of PD-1-Y248 is a marker of PD-1-mediated inhibitory function in human T cells
Source: Sci Rep. 2019 Nov 21;9:17252. doi: 10.1038/s41598-019-53463-0 (PMC6872651; doi:10.1038/s41598-019-53463-0)
Supplement: Supplementary file 1 — Supplementary Figures [file 41598_2019_53463_MOESM1_ESM.pdf]

Phosphorylation of PD-1-Y248 is a marker of PD-1-mediated inhibitory function in human T cells

Supplementary Materials

Kankana Bardhan[1,2#], Halil-Ibrahim Aksoylar[1,2#], Thibault Le Bourgeois[1,2^], Laura Strauss[1,2], Jessica D. Weaver[1,2], Bethany Delcuze[2], Alain Charest[2,3], Nikolaos Patsoukis[1,2], Vassiliki A. Boussiotis[1,2,3]*

[#]First co-authors

^Current address: Antoine Lacassagne Cancer Institute of Nice, Medical University of Nice Sophia Antipolis

[1]Division of Hematology-Oncology, [2]Department of Medicine and [3]Cancer Center, Beth Israel Deaconess Medical Center, Harvard Medical School, Boston MA 02215

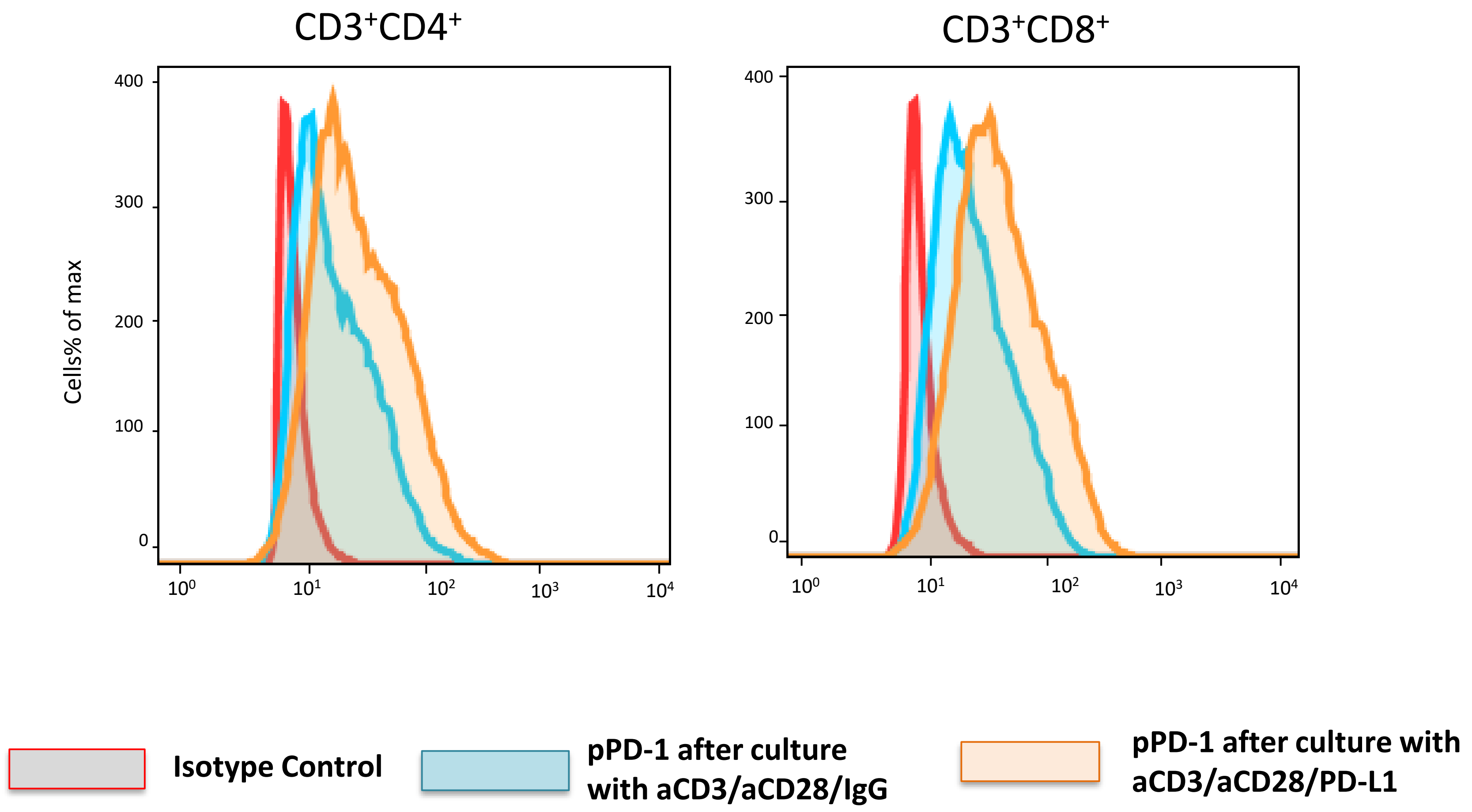

Figure S1: PD-1 phosphorylation is induced by aCD3/aCD28 stimulation and is enhanced by PD-1 co-ligation with PD-L1. Purified T cells were cultured for 48 hours with aCD3/aCD28/IgG or aCD3/aCD28/PD-L1-Ig, as described in methods, and after gating on $CD4^+$ or $CD8^+$ T cells expression of pPD-1 was examined. Overlay graphs of the data shown in Figure 2B and D.

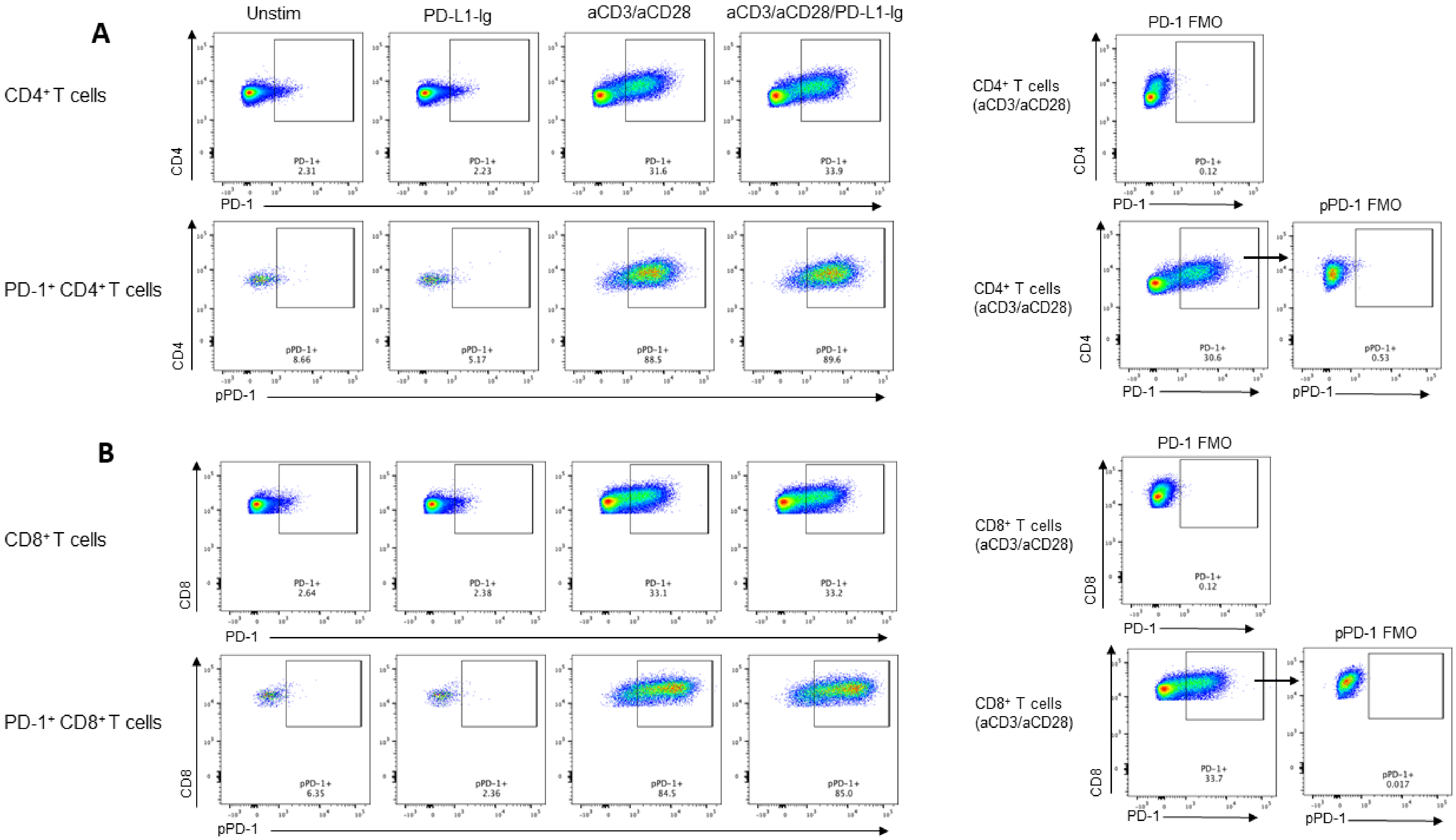

Figure S2: CD4+ and CD8+ T cells were either left unstimulated or cultured with PD-L1-Ig, aCD3/aCD28, or aCD3/aCD28/PD-L1-Ig, as indicated in Methods, and expression of PD-1 was assessed by flow cytometry. In each culture condition, after gating in PD-1^{+} cells, expression of pPD-1 was assessed. Fluorescence minus one (FMO) staining controls for PD-1 and pPD-1 were assessed for CD4^{+} and CD8+ T cells cultured with aCD3/aCD28 mAbs.

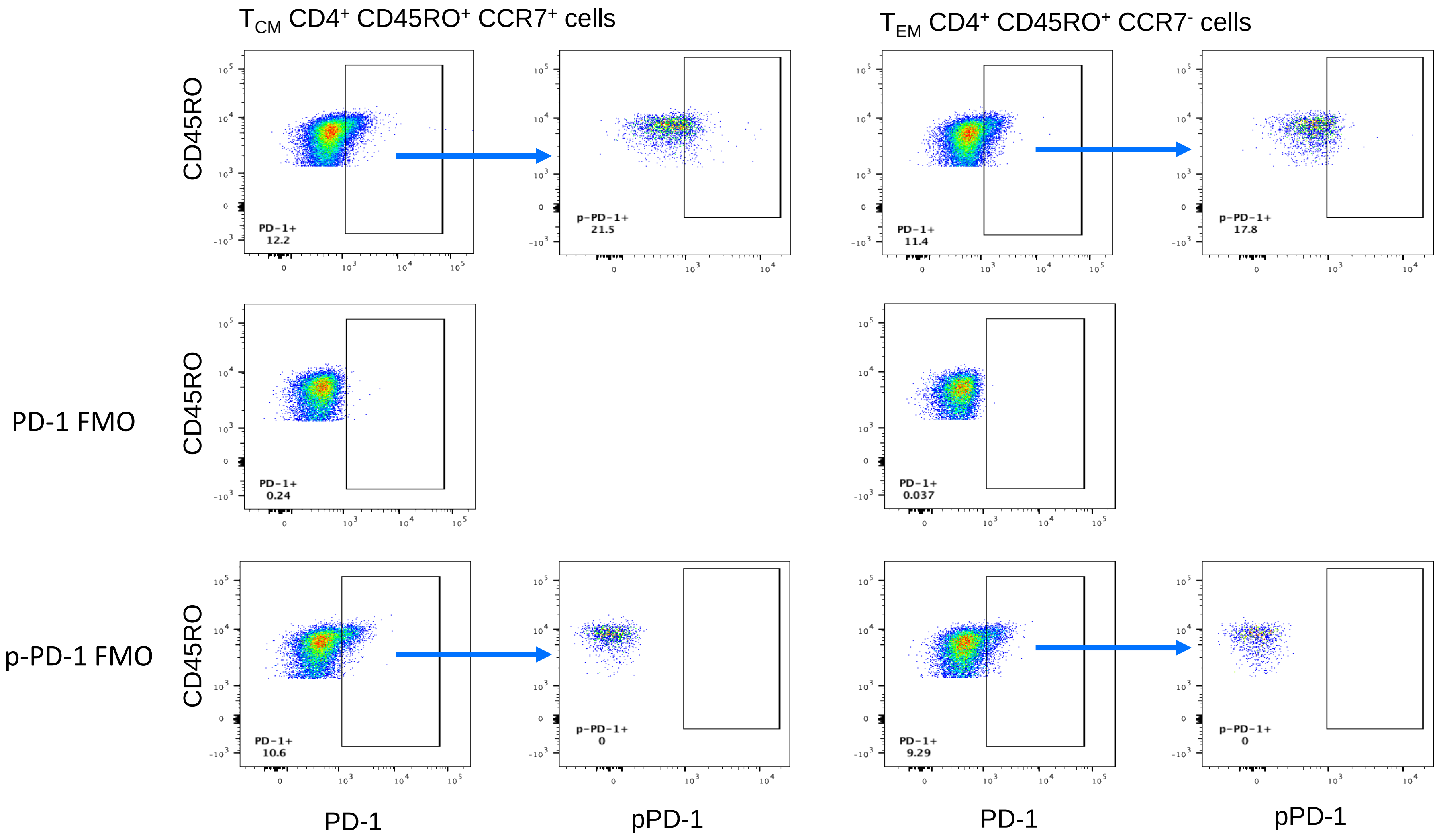

Figure S3A: Fluorescence minus one (FMO) staining controls for PD-1 and p-PD-1 were assessed for CD4+ T_{EM} and T_{CM} cells. CD4+T T_{EM} and T_{CM} cells were identified as in Figure 3. For PD-1 FMO, cells were labeled with all antibodies excluding PD-1 while for the pPD-1 FMO, pPD-1 antibody is excluded.

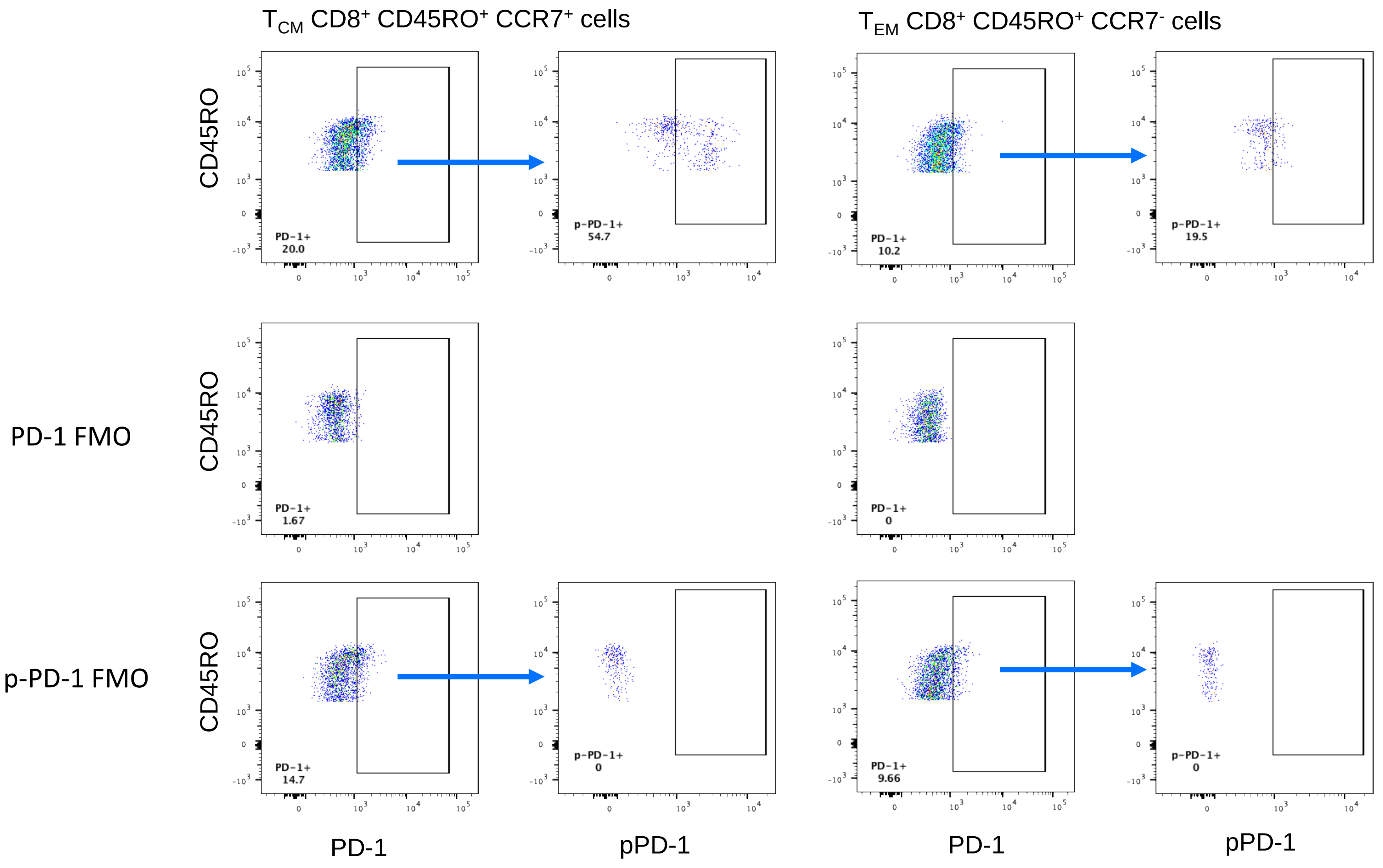

Figure S3B: Fluorescence minus one (FMO) staining controls for PD-1 and pPD-1 were assessed for CD8$^+$ T_{EM} and T_{CM} cells as performed in Figure S3A.
